# Diabetes Prevalence and Control Among US Adults

**DOI:** 10.1001/jamanetworkopen.2026.40877

**Published:** 2026-10-28

**Authors:** Jonathan Skinner, Bradley White, Tracy Hammonds, Partha Bhattacharyya, Mir M. Ali

**Affiliations:** 1 Office of Data Resources and Analytics, Division of Behavioral and Social Research, National Institute on Aging, National Institutes of Health, Bethesda, Maryland; 2 National Bureau of Economic Research, Cambridge, Massachusetts; 3 Real-Word Data Insights, HealthVerity, Inc, Philadelphia, Pennsylvania; 4 Office of the Chief Information Officer, US Department of Health and Human Services, Washington, DC

## Abstract

This cross-sectional study examines the prevalence of diabetes, hyperglycemia, and hypoglycemia among US adults based on coded diagnoses in a medical claims dataset.

## Introduction

Rates of diabetes and other chronic diseases have been rising during the last several decades, increasing concerns about the current and future health of US residents.^1^ Previous studies have found increases in overall US diabetes rates from 9.8% to 14.3% between 1999 and 2018,^2^ increases in elevated glycemic levels,^3^ and increased diabetes prevalence through 2023.^4^ However, the modest sample sizes in these studies preclude granular analysis of small geographic areas, and no recent evidence is available on rates of diagnosed hyperglycemia or hypoglycemia. We therefore estimated the prevalence of coded diagnoses of diabetes, hyperglycemia, and hypoglycemia in persons aged 18 to 64 years during 2018 to 2024 using a multipayer adjudicated medical claims dataset.

## Methods

In this cross-sectional study, we analyzed a Health Insurance Portability and Accountability Act–compliant, deidentified claims dataset maintained and licensed by HealthVerity, Inc. The study followed the Strengthening the Reporting of Observational Studies in Epidemiology (STROBE) reporting guideline. The National Institutes of Health Institutional Review Board deemed the study exempt from review and waived the informed consent requirement because it used deidentified secondary data.

Data were restricted to closed claims for 12 months (or months until death), meaning all patient claims are available. At least 2 outpatient diagnoses of diabetes (*International Classification of Diseases, Tenth Revision* codes E8 to E11 or E13) more than 7 days apart or 1 inpatient diagnosis was required for a diabetes diagnosis.^5^ The prevalence rate of diabetes with hyperglycemia (E10.65 or E11.65) or hypoglycemia codes (E10.64 or E11.64) was defined as the number of persons with at least 1 diagnosis during the year divided by the total number of persons diagnosed with diabetes; all measures were adjusted by age and sex. Additionally, prevalence rates were estimated for the US by sex, insurance coverage (commercial insurance, Medicare Advantage coverage for persons aged <65 years, and Medicaid managed care), and state.

Data were analyzed between November 2025 and January 2026 using Stata, version 19 (StataCorp LLC) and R, version 4.4.1 (R Project for Statistical Computing). We report 2-sided 95% CIs.

## Results

The sample included 285 213 216 person-years (median [range] age, 41 [18-64] years; 54.8% female) of data (Table reports demographics). The mean prevalence rate of diabetes rose from 8.99% in 2018 to 10.61% in 2024, an increase of 1.63 percentage points (pp) (95% CI, 1.62-1.64 pp); similar patterns were seen for men and women. Although the prevalence rates among commercial insurance enrollees were lower than among Medicaid and Medicare beneficiaries, the growth in prevalence in the former group was the most rapid (from 7.49% to 9.46%, an increase of 1.97 pp [95% CI, 1.96-1.98 pp]). Overall, the prevalence rate of diagnosed hyperglycemia (conditional on having diabetes) during 2018 to 2024 increased from 33.96% to 37.32%, rising 3.36 pp (95% CI, 3.29-3.42 pp); there was little change in the rate of diagnosed hypoglycemia.

**Table.** Prevalence Rates of Diagnosed Diabetes, Hyperglycemia, and Hypoglycemia by Year and Demographic Characteristics in the US, 2018 to 2024^a^

Characteristic	Mean No. of persons per y, millions^b^	Prevalence rate (95% CI), %			Change during 2018-2024 (95% CI), percentage points
		2018	2021	2024	
Annual sample, millions^c^	NA	44.88	38.74	33.14	NA
Diabetes prevalence	40.75	8.99 (8.98 to 9.00)	9.93 (9.92 to 9.94)	10.61 (10.60 to 10.62)	1.63 (1.62 to 1.64)
Women	23.52	7.83 (7.82 to 7.84)	8.80 (8.79 to 8.81)	9.37 (9.35 to 9.38)	1.53 (1.52 to 1.56)
Men	17.23	10.56 (10.55 to 10.58)	11.47 (11.46 to 11.49)	12.31 (12.30 to 12.33)	1.75 (1.74 to 1.76)
Commercial insurance	28.20	7.49 (7.48 to 7.49)	8.18 (8.17 to 8.19)	9.46 (9.44 to 9.47)	1.97 (1.96 to 1.98)
Managed Medicaid	12.21	14.08 (14.06 to 14.10)	14.10 (14.08 to 14.12)	14.30 (14.28 to 14.33)	0.22 (0.20 to 0.24)
Medicare Advantage	0.31	20.32 (20.20 to 20.43)	20.96 (20.80 to 21.10)	21.21 (21.01 to 21.40)	0.90 (0.74 to 1.03)
Diagnosed hyperglycemia^d^	3.99	33.96 (33.91 to 34.00)	36.27 (36.22 to 36.32)	37.32 (37.27 to 37.37)	3.36 (3.29 to 3.42)
Women	2.02	33.50 (33.43 to 33.56)	35.64 (35.58 to 35.71)	36.48 (36.41 to 36.55)	2.98 (2.89 to 3.07)
Men	1.97	34.43 (34.37 to 34.40)	36.91 (36.84 to 36.91)	40.47 (40.40 to 40.55)	3.82 (3.65 to 3.84)
Commercial insurance	2.50	33.38 (33.33 to 33.44)	35.66 (35.59 to 35.72)	37.37 (37.31 to 37.43)	3.99 (3.91 to 4.07)
Managed Medicaid	1.39	35.21 (35.12 to 35.29)	37.35 (37.27 to 37.43)	37.36 (37.27 to 37.45)	2.15 (2.04 to 2.27)
Medicare Advantage	0.06	35.40 (35.15 to 35.65)	37.37 (37.06 to 37.67)	36.98 (36.58 to 37.39)	1.60 (1.14 to 2.02)
Diagnosed hypoglycemia^d^	3.99	2.48 (2.46 to 2.49)	2.72 (2.70 to 2.74)	2.47 (2.46 to 2.49)	0.00 (−0.01 to 0.01)
Women	2.02	2.63 (2.60 to 2.65)	2.81 (2.78 to 2.83)	2.52 (2.50 to 2.54)	−0.07 (−0.14 to 0.07)
Men	1.97	2.32 (2.30 to 2.34)	2.63 (2.61 to 2.66)	2.43 (2.40 to 2.45)	0.10 (0.07 to 0.13)
Commercial insurance	2.50	2.12 (2.11 to 2.14)	2.28 (2.26 to 2.30)	2.17 (2.16 to 2.19)	0.05 (0.02 to 0.07)
Managed Medicaid	1.39	3.11 (3.08 to 3.14)	3.38 (3.35 to 3.41)	3.14 (3.10 to 3.17)	0.02 (−0.02 to 0.06)
Medicare Advantage	0.06	4.58 (4.47 to 4.69)	4.22 (4.09 to 4.35)	4.86 (4.68 to 5.04)	0.29 (0.09 to 0.47)

Abbreviation: NA, not applicable.

^a^ All measures (except for sample count means) are reported in percentage terms.

^b^ Sample of persons diagnosed with diabetes. Diabetes prevalence used to estimate hyperglycemia and hypoglycemia prevalence.

^c^ Sample of all enrollees used to estimate diabetes prevalence; total 285 213 216 person-years of data.

^d^ Percentages of diagnosed hypoglycemia and hyperglycemia are relative to the population of persons diagnosed with diabetes. Sample declines during the period because of loss of an insurance source.

The Figure shows state-specific age- and sex-adjusted rates of diabetes prevalence, diagnosed hyperglycemia, and diagnosed hypoglycemia, with the 2 states with the highest and lowest mean rates over the study period highlighted. The general lack of increase in diabetes prevalence between 2019 and 2020 is most likely the consequence of reduced health care utilization during the COVID-19 pandemic.^6^ There were regional differences in diabetes prevalence rates in all years; in 2024, rates ranged from 6.01% (95% CI, 5.89%-6.13%) in Montana to 14.88% (95% CI, 14.74%-15.03%) in Mississippi, but all states showed an upward trend over time. Prevalence rates of diagnosed hyperglycemia exhibited similar degrees of geographic variation, from 20.43% (95% CI, 20.00%-20.85%) in Colorado to 44.71% (95% CI, 44.38%-44.05%) in Arizona in 2024. There was little variation in diagnosed hypoglycemia rates.

**Figure.**
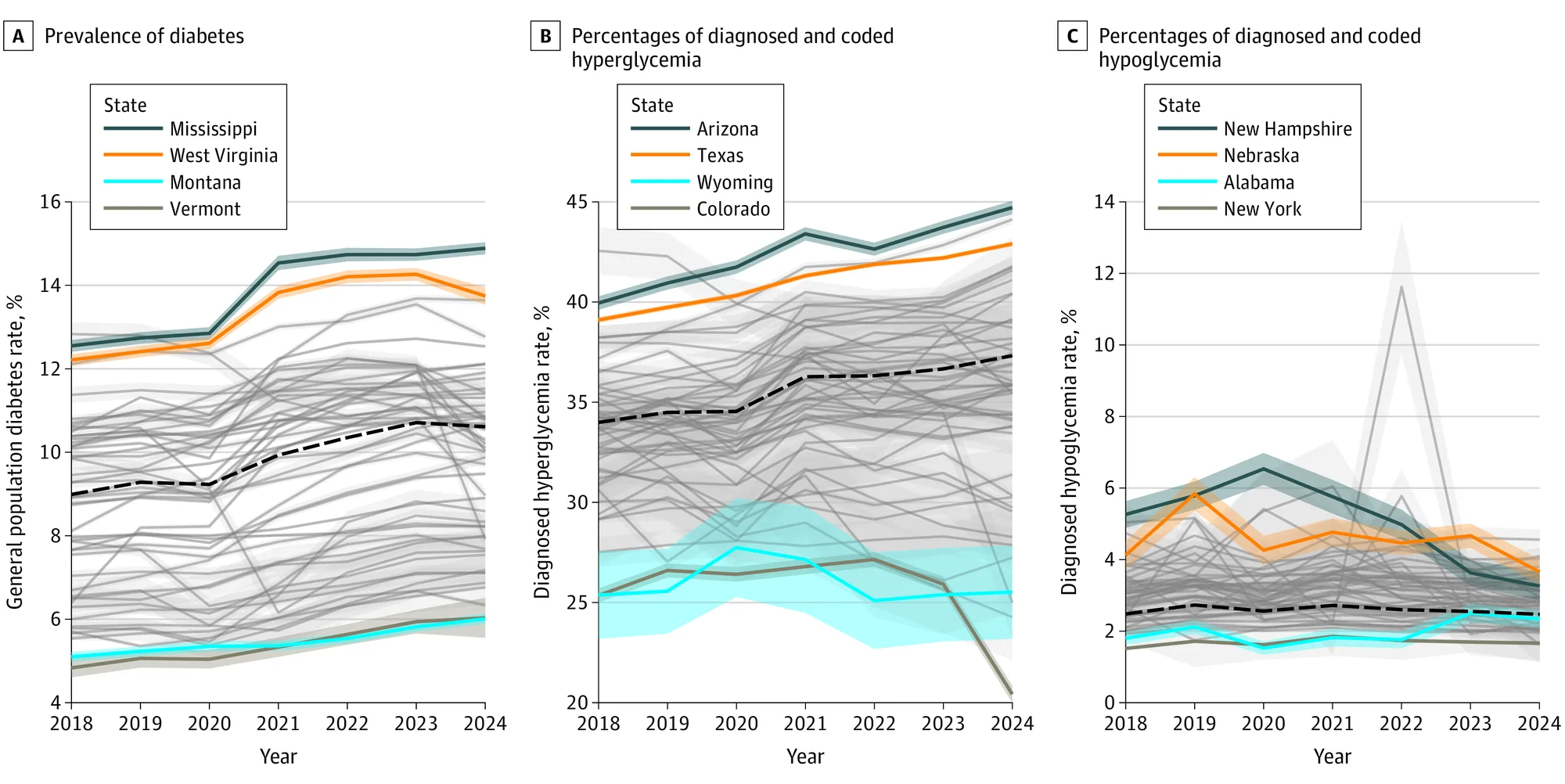
Line Graphs Showing Prevalence Rates for Diagnosed Diabetes, Hyperglycemia, and Hypoglycemia in the US, 2018 to 2024 A, Prevalence rates of diabetes in the general population by state. Data are age- and sex-adjusted rates of diagnosed diabetes in the population aged 18 to 64 years. B, Percentage of diagnosed and coded hyperglycemia presented as a percentage of persons diagnosed with diabetes. C, Percentage of diagnosed and coded hypoglycemia presented as a percentage of persons diagnosed with diabetes; the outlier in 2022 (11.62%) is Rhode Island. Shading indicates 95% CIs. Colored lines indicate the 2 states with the highest rates (dark blue and orange) and lowest rates (light blue and brown) over the study period. The black dashed line indicates the US mean.

## Discussion

Prevalence rates of both diagnosed diabetes and hyperglycemia increased for US residents aged 18 to 64 years during 2018 to 2024, whether by sex or insurance coverage. We also found variations in rates of diagnosed diabetes and hyperglycemia across states, suggesting an important role for linked data in identifying geographic hot spots.

This study has several limitations. First, the measures reflect diagnosed diabetes rather than true prevalence; upward trends may reflect an increase in the detection of previously undiagnosed diabetes. However, Gwira et al^4^ reported that the trend in diagnosed diabetes was the consequence of true diabetes growth rather than a reduction in undiagnosed diabetes. Second, variations in coded hyperglycemia may be the consequence of differences in screening and billing practices across insurance plans or over time. Third, 1 commercial insurance plan was dropped from the dataset in 2024; several states experienced a decline in their sample size as a result. Therefore, some variations observed in 2024 could be the consequence of a different mix of enrollees in the state. Finally, we cannot determine why states differed so much in the prevalence of diabetes and rates of diagnosed hyperglycemia.

In conclusion, we found large geographic differences in the prevalence of diagnosed diabetes and diagnosed hyperglycemia, as well as an upward trend in rates for women and men and across insurance groups. Large-scale claims data represent a promising approach to documenting and monitoring efforts to reduce prevalence rates of chronic disease in the adult population.

## Notes

Supplement.
Data Sharing Statement
