# Supplementary material for: Diabetes Prevalence and Control Among US Adults
Source: JAMA Netw Open. 2026 Oct 28;9(10):e2640877. doi: 10.1001/jamanetworkopen.2026.40877 (PMC13717414; doi:10.1001/jamanetworkopen.2026.40877)
Supplement: Supplementary file 1 [file jamanetwopen-e2640877-s001.pdf]

Data Sharing Statement

Skinner. Diabetes Prevalence and Control Among US Adults. *JAMA Netw Open*. Published October 28, 2026. doi:10.1001/jamanetworkopen.2026.40877

Data
Data available: No
